# 2-Hydroxybenzophenone Derivatives: ESIPT Fluorophores Based on Switchable Intramolecular Hydrogen Bonds and Excitation Energy–Dependent Emission

**DOI:** 10.3389/fchem.2021.766179

**Published:** 2021-10-19

**Authors:** Hailan Wang, Yuxin Xiao, Zongliang Xie, Haodong Sun, Xiayu Zhang, Juan Wang, Rongjuan Huang

**Affiliations:** ^1^ Frontiers Science Center for Flexible Electronics (FSCFE), Shaanxi Institute of Flexible Electronics (SIFE) and Shaanxi Institute of Biomedical Materials and Engineering (SIBME), Northwestern Polytechnical University (NPU), Xi’an, China; ^2^ School of Packaging and Materials Engineering, Hunan University of Technology, Zhuzhou, China

**Keywords:** ESIPT, dual-emission, tunable color, Enol form, Keto form, excitation dependence

## Abstract

In this work, a new series of 2-hydroxybenzophenone (BPOH) derivatives, BPOH-TPA, BPOH-PhCz, and BPOH-SF substituting with different electron-donating groups are designed and synthesized. Dual-emission spectra are observed in solutions indicating their excited-state intramolecular proton transfer (ESIPT) character. In solid states, all compounds exhibit a broad emission spectrum when excited at low excitation energy, deriving from the enol-type form stabilized by intramolecular hydrogen bonds. Compound BPOH-TPA shows a clear excitation wavelength dependence. However, such behavior is absent in BPOH-PhCz and BPOH-SF, as the rigid and weaker donor moieties may restrict this process. Furthermore, by increasing the excitation energy, dual emission with a high-energy band ranging from 550 to 582 nm and a low-energy band ranging from 625 to 638 nm is obtained in all three molecules. The photophysical studies and single-crystal analyses are performed to further illustrate the excitation-dependent emission. Higher excitation energies can promote more excitons to keto forms via ESIPT, giving a stronger redshifted emission. BPOH-TPA with a stronger donor strength exhibits an obvious color change gradually from yellow to orange-red with the increasing excitation power from 1 to 15 mW/cm^2^. This study provides a novel example of ESIPT materials with tunable emission colors.

## Introduction

Metal-free organic materials with dual emission have raised particular attention for their great potential applications in areas of anti-counterfeiting (Yang et al., 2019; Huang et al., 2020), sensing (Li et al., 2005; Vendrell et al., 2012; Zhu et al., 2015; Chen et al., 2016; Jin et al., 2020), biological imaging (Chu et al., 2016; Sun et al., 2016; Mulay et al., 2018), and single-component white organic light-emitting devices (WOLEDs) (Baldo et al., 1998; Lamansky et al., 2001; Su et al., 2006; Chan et al., 2012; Shao et al., 2012; Xie et al., 2017). However, according to Kasha’s rule, molecules only emit from the lowest-energy excited electronic state of a given multiplicity (Kasha, 1950), giving only one emission band. The development of dual-emissive materials is of great significance to both the industrial arena and scientific challenges (Yanagi and Kataura, 2010; Demchenko et al., 2017).

**FIGURE 1:**
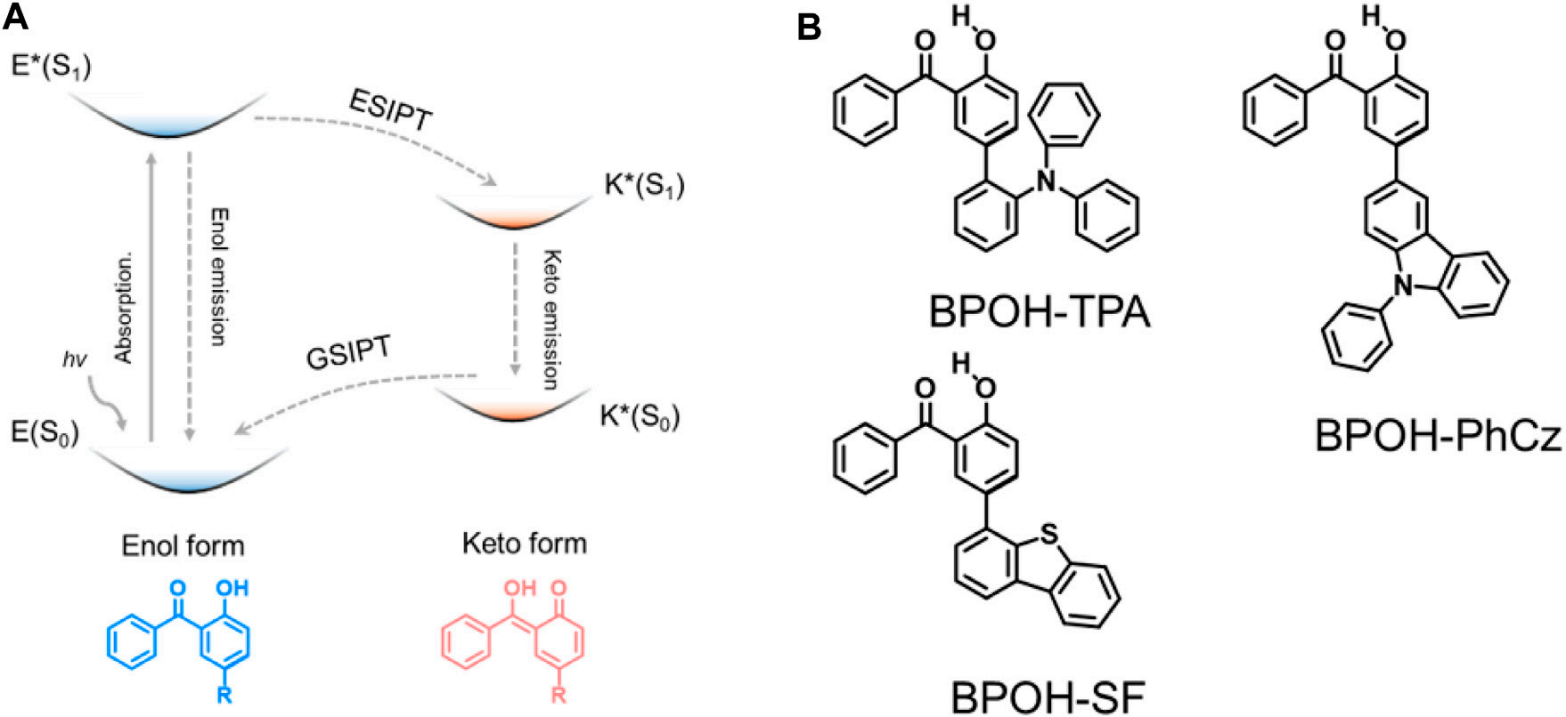
**(A)** Schematic of the ESIPT mechanism and **(B)** chemical structures of BPOH-TPA, BPOH-PhCz, and BPOH-SF.

To achieve dual emission, a series of strategies have been reported, such as by forming excimers (Lee et al., 2014; Chen et al., 2016), combining intra- and intermolecular charge transfer (Li et al., 2019; Wen et al., 2020), and constructing supramolecular self-assembles (Pal et al., 2017; Li et al., 2018; Wang et al., 2020) and excited-state intramolecular proton transfer (ESIPT) emission (Sakai et al., 2016; He et al., 2018; Li et al., 2018; Sedgwick et al., 2018; Suzuki et al., 2018; Wu et al., 2018). As a good approach that can rationally tune the dual-emission property, ESIPT involves a rapid photoinduced tautomerization of a molecule in its electronic excited state, in which the protons are prone to transfer to adjacent heteroatoms through intramolecular hydrogen bonding forming excited-state isomers, i.e., from the enol (E*) to keto (K*) formula (Figure 1A). Subsequent emission occurs from one tautomer or both tautomers giving one or dual emission. For different excited-state isomers, the excited-state relaxation processes and charge distributions are quite distinct to realize dual-emissive properties. The ESIPT process can potentially induce a significant change in the electron density distribution of the frontier molecular orbitals between the E* and K* forms, leading to a change in the singlet-triplet energy gap.

Many researches based on ESIPT materials have been published, such as sensing and white OLEDs. For instance, Yang and coworkers have developed a dual-emissive ESIPT material N-salicylidene-3-hydroxy-4-(benzo[d]thiazol-2-yl) phenylamine (Sun et al., 2009). In 2016, Chou and coworkers designed a new ESIPT molecule, t-MTTH with thiazolo[5,4-d]thiazole nucleus, as a proton receptor (Zhang et al., 2016). The dual emission derived from the normal (ca. 440 nm) and isomer (ca. 560 nm) almost covers the entire visible region. White OLED based on this material was successfully fabricated with the CIE coordinates of (0.29, 0.33). Although some represented ESIPT materials have been investigated, the library of ESIPT molecules and the mechanism are still insufficient. The development of new ESIPT molecules with simple chemical structures and high performance is still of great challenge. Besides, the manipulation of the emission colors of the dual-emissive ESIPT materials is also very attractive for smart materials. In 2-hydroxybenzophenone (BPOH), the intermolecular O−H···O hydrogen bond could be formed between the carbonyl group of the benzophenone moiety and the neighboring phenolic hydroxyl group (Tang et al., 2011). Along with the photoinduced formation of the O−H···O hydrogen bonds, the protons of the phenolic hydroxyl group can also be easily transferred to the carbonyl group in excited states. Thus, the K* form of BPOH moiety could be generated. The proton accepting ability can be significantly increased by tailoring an electron-donating group based on hydrogen bonding. Tailoring three substituents with different electron donating abilities was carried out for BPOH to probe the ESIPT process.

**SCHEME 1:**
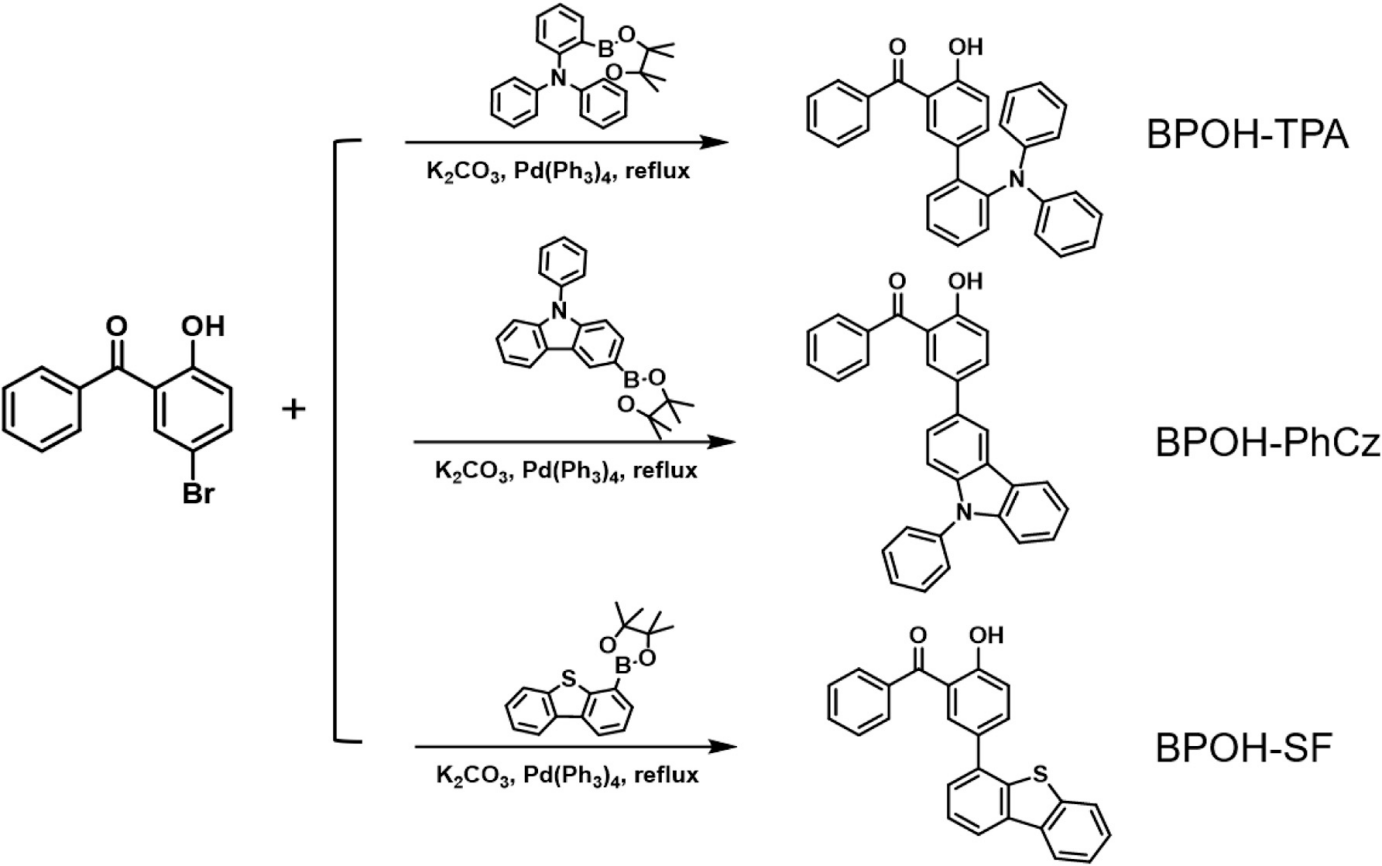
Synthetic routes of compounds BPOH-TPA, BPOH-PhCz and BPOH-SF.

In this work, we designed and synthesized three donor–acceptor-type materials, 5-(2-triphenylamine)-2-hydroxybenzophenone (BPOH-TPA), 5-(9-phenyl carbazole)-2-hydroxybenzophenone (BPOH-PhCz), and 5-(4-dibenzothiophene)-2-hydroxybenzophenone (BPOH-SF), by employing BPOH as the acceptor unit (Figure 1B). The detailed synthetic processes are shown in Scheme 1. The dual-emission is mainly attributed to the intramolecular proton transfer of the hydrogen bond in the 2-BPOH acceptor. Photophysical studies and single-crystal analyses were carried out to confirm the origin of dual-emission. Additionally, all three compounds exhibit excitation energy–dependent emissions in a solid state. By increasing the optical power densities of excitation resource from 1 to 15 mW/cm^2^, the ratio of the low-energy band to the high-energy band increases gradually with a color change from yellow to orange-red. This study provides a novel strategy for designing ESIPT materials with tunable emission colors.

## Materials and Methods

### Methods

^1^H NMR and ^13^C NMR spectra were performed on a Bruker Avance Neo 500 Nuclear Magnetic Resonance Spectrometer. High-Resolution EI mass spectra were measured with an Exactive GC high-resolution mass spectrometer. The UV-visible absorption spectra were carried out with a Hitachi U-3900 spectrophotometer. The excitation, steady-state, and time-resolved spectra were measured in Edinburgh FLS1000 fluorescence spectrophotometer equipped with a laser light source (295 nm). The emission spectra of these compounds with different optical power densities of excitation were carried out on Ocean Optics QE65 pro with 365 nm THORLABS LED as an excitation source. Single-crystal analyses of BPOH-TPA were determined using an Oxford Diffraction Gemini S Ultra X-ray Single-Crystal Diffractometer with a (Cu) X-ray source.

### Syntheses

The synthetic route to the three compounds (5-(2-triphenylamine)-2-hydroxybenzophenone (BPOH-TPA), 5-(9-phenyl carbazole)-2-hydroxybenzophenone (BPOH-PhCz), and 5-(4-dibenzothiophene)-2-hydroxybenzophenone (BPOH-SF)) is shown in Scheme 1. BPOH-TPA, BPOH-PhCz, and BPOH-SF were obtained in a good yield by the Suzuki coupling reaction of 5-bromo-2-hydroxybenzophenone with 2-(diphenylamino)-benzeneboronic acid pinacol ester, 9-phenyl-3-(4,4,5,5-tetramethyl-1,3,2-dioxaborolan-2-yl) carbazole, and 4-(4,4,5,5-tetramethyl-1,3,2-dioxaborolan-2-yl)dibenzothiophene, respectively. The chemical structures of the final compounds were confirmed by ^1^H NMR, ^13^C NMR, and high-resolution EI mass spectra in the Supplementary Material (Supplementary Figures S1–S3).

#### 5-(2-Triphenylamine)-2-hydroxybenzophenone

A mixture of 5-bromo-2-hydroxybenzophenone (1.50 g, 5.45 mol), 2-(diphenylamino)benzeneboronic acid pinacol ester (2.21 g, 5.95 mol), potassium carbonate (2.24 g, 16.24 mol), and distilled water (10 ml) was dissolved in 50 ml degassed tetrahydrofuran (THF) under nitrogen atmosphere. Then, Pd(PPh_3_)_4_ (0.10 g) was added to the mixture under a nitrogen atmosphere. After reflexing for 16 h, the mixture was extracted with dichloromethane and dried with sodium sulfate anhydrous. It was further purified through silica-gel column chromatography with dichloromethane/hexane (1/2, v/v) as eluent to give BPOH-TPA as a yellow powder (0.70 g, yield: 29.29%). ^1^H NMR (500 MHz, DMSO-*d*_6_): δ = 10.35 (s, 1H), 7.62–7.57 (m, 1H), 7.46 (d, *J* = 4.4 Hz, 4H), 7.39 (td, *J* = 7.5, 1.9 Hz, 1H), 7.36–7.28 (m, 3H), 7.18–7.09 (m, 6H), 6.86 (t, *J* = 7.3 Hz, 2H), 6.79 (d, *J* = 8.5 Hz, 1H), 6.75 (d, *J* = 7.9 Hz, 4H). High-Resolution EI MS: m/z found: 441.1722 [M]^+^; calcd for C_31_H_23_NO_2_: 441.1729.

#### 5-(9-Phenyl carbazole)-2-hydroxybenzophenone

Compound BPOH-PhCz was achieved according to the aforementioned procedure for BPOH-TPA with 9-phenyl-3-(4,4,5,5-tetramethyl-1,3,2-dioxaborolan-2-yl)carbazole (2.20 g, 5.95 mmol) in place of 2-(diphenylamino)benzeneboronic acid pinacol ester. Yield: 1.85 g (78.81%). ^1^H NMR (500 MHz, DMSO-*d*_6_): δ = 10.34 (s, 1H), 8.52 (d, *J* = 1.9 Hz, 1H), 8.33 (dt, *J* = 7.8, 1.0 Hz, 1H), 7.89–7.79 (m, 3H), 7.72–7.62 (m, 7H), 7.55 (td, *J* = 7.5, 3.8 Hz, 3H), 7.46–7.36 (m, 3H), 7.29 (ddd, *J* = 8.0, 7.0, 1.1 Hz, 1H), 7.11 (d, *J* = 8.5 Hz, 1H). High-Resolution EI MS: m/z found: 439.1524 [M]^+^; calcd for C_31_H_21_NO_2_: 439.1572.

#### 5-(4-Dibenzothiophene)-2-hydroxybenzophenone

Compound BPOH-SF was achieved according to the aforementioned procedure for BPOH-TPA with 4-(4,4,5,5-tetramethyl-1,3,2-dioxaborolan-2-yl)dibenzothiophene (1.85 g, 5.95 mmol) in place of 2-(diphenylamino)benzeneboronic acid pinacol ester. Yield: 1.75 g (84.98%). ^1^H NMR (500 MHz, DMSO-*d*_6_): δ =10.64 (s, 1H), 8.45–8.32 (m, 2H), 8.08–8.00 (m, 1H), 7.88–7.80 (m, 3H), 7.72 (d, *J* = 2.4 Hz, 1H), 7.69–7.53 (m, 7H), 7.20 (d, *J* = 8.5 Hz, 1H). High-Resolution EI MS: m/z found: 380.0857 [M]^+^; calcd for C_25_H_16_O_2_S: 380.0871.

## Results and Discussion

### Photophysical Characterization

**FIGURE 2:**
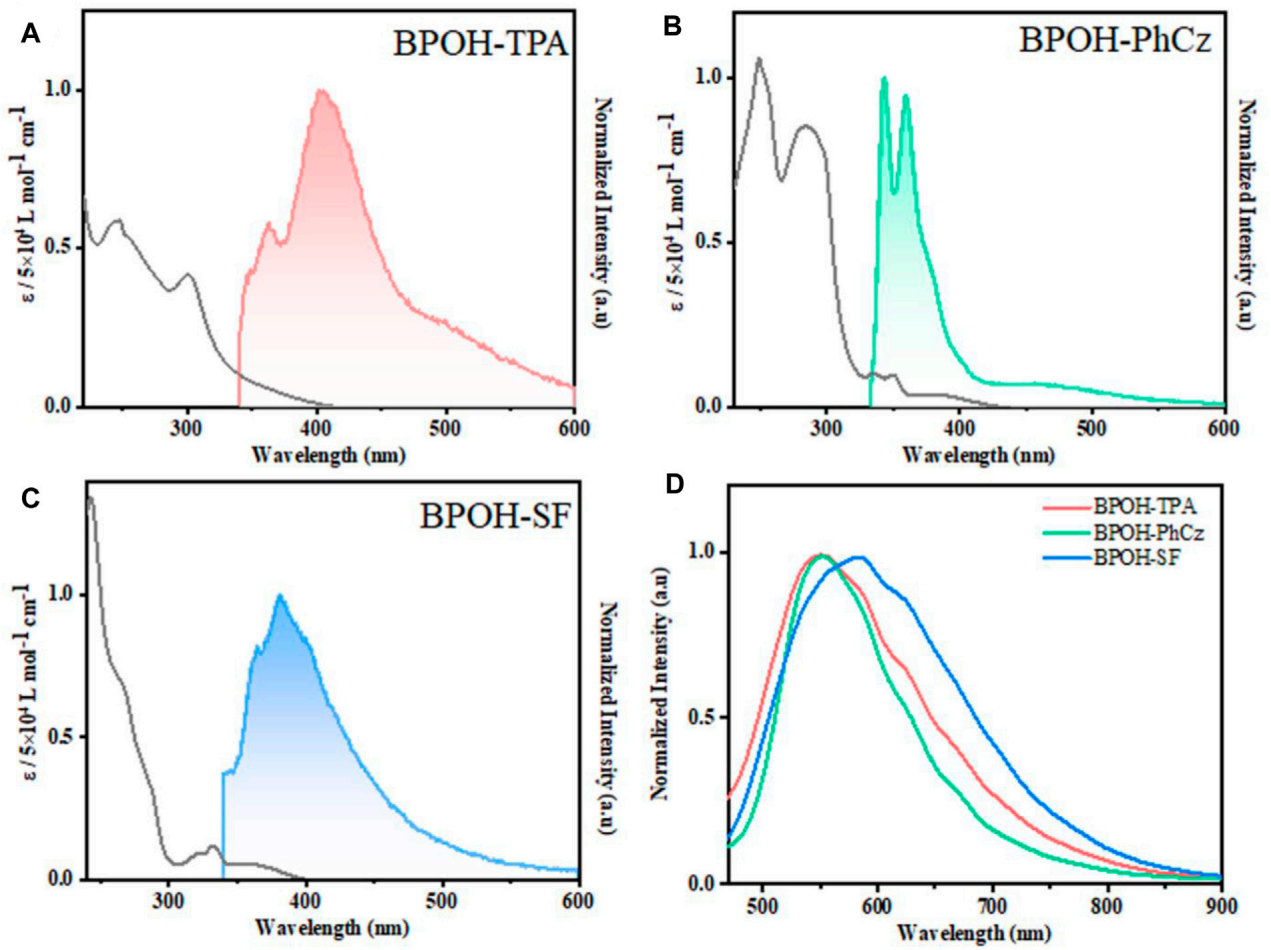
Absorption (black) and fluorescence spectra of **(A)** BPOH-TPA, **(B)** BPOH-PhCz, and **(C)** BPOH-SF in THF at a concentration of 2.0 × 10^−5^ mol L^−1^ (*λ*_ex_ = 295 nm). **(D)** The emission spectra of BPOH-TPA, BPOH-PhCz, and BPOH-SF in powder states (*λ*_ex_ = 365 nm).

The photophysical properties of BPOH-TPA, BPOH-PhCz, and BPOH-SF were investigated in both solution and powder states. The absorption and emission spectra of all molecules in diluted THF solutions are shown in Figures 2A–C. The high-energy absorption bands below 300 nm and lower-energy transitions in the range of 318–400 nm can be ascribed to the π-π* transitions from enol and keto forms, as the absorption spectra are independent of the solvent polarity (Supplementary Figure S5). This can be further verified in steady-state emission measurement. All the measurements were conducted in dilute solutions (n-hexane, THF, MeOH solution, 2.0 × 10^−5^ mol L^−1^), which confirms that the lower-energy absorption bands did not originate from molecular aggregations. In molecules BPOH-TPA and BPOH-PhCz, well-structured dual-emission spectra are observed, indicating their ESIPT character. The high-intensity bands at the high energy are assigned as the emission of enol form, whereas the low-energy emission tails ranging from 464 to 518 nm are attributed to the keto form. In contrast, a different emission behavior is observed in the molecule BPOH-SF. It is proposed that the emission from the keto form of BPOH-SF is too weak compared to that of the enol form. Analyzing the lifetime decays of the enol forms in these three compounds are performed in THF solutions (Supplementary Figures S6–S8). They all exhibit short lifetimes of a few nanoseconds, confirming the fast ESIPT process. In the solid state, the fluorescence spectra of all compounds show similar broad shapes at low excitation energy (1 mW/cm^2^), deriving from the enol-type form stabilized by intramolecular hydrogen bonds. With the increasing donor strength, BPOH-TPA, BPOH-PhCz, and BPOH-SF show redshifted emission with the peak wavelength at 550, 558, and 582 nm, respectively (Figure 2D). Moreover, BPOH-TPA, BPOH-PhCz, and BPOH-SF exhibit relatively low photoluminescence quantum yields (Φ_PL_), which can be ascribed to the extremely fast proton transfer process from the E* to K* formula (Table 1).

**TABLE 1:** Photophysical properties of BPOH-TPA, BPOH-PhCz, and BPOH-SF in solution and solid states at room temperature.

Compounds	*λ*_abs_/nm^a^	*λ*_em_/nm^a^	ε/L mol^−1^cm^−1^ ^a^	*τ* (ns)/λ (nm)^a^	Φ_PL_(%)^b^
BPOH-TPA	243, 299	402, 518	0.294 × 10^5^	3.26/405	0.256
BPOH-PhCz	248, 283, 348	344, 464	0.527 × 10^5^	7.38/343	0.297
BPOH-SF	258, 332, 362	382, 487	0.670 × 10^5^	2.31/382	0.375

a In THF solution (2.0 × 10^−5^ mol L^−1^).

b In powder state.

**FIGURE 3:**
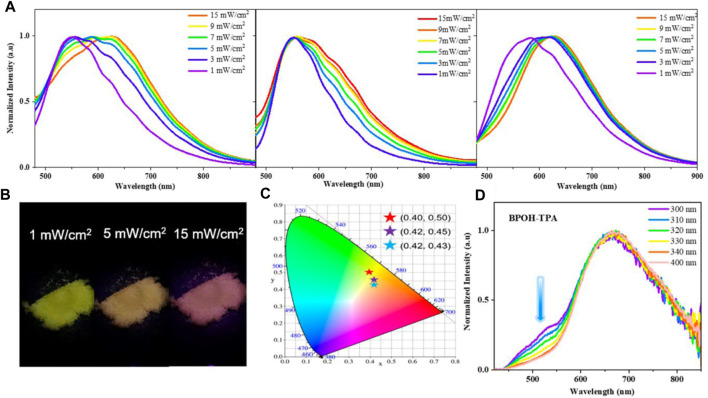
**(A)** Dependence of the emission spectra of BPOH-TPA, BPOH- PhCz, and BPOH-SF upon different power densities (1–15 mW/cm^2^), *λ*_ex_ = 365 nm. **(B)** The photographs of BPOH-TPA powder taken under the excitation power of 1, 5, and 15 mW/cm^2^, with the **(C)** CIE 1931 chromaticity coordinates of (0.40, 0.50), (0.42, 0.45), and (0.42, 0.43), respectively. **(D)** Emission spectra of BPOH-TPA upon different excitation wavelengths (300−400 nm).

To further explore the ESIPT character, the influence of excitation energy on the emission spectra of molecules BPOH-TPA, BPOH-PhCz, and BPOH-SF in solid states was investigated. As shown in Figure 3A, a clear redshift is observed in the emission spectra of all three compounds BPOH-TPA, BPOH-PhCz, and BPOH-SF, by gradually increasing the optical power densities of excitation resources from 1 to 15 mW/cm^2^. Taking BPOH-TPA as an example, an emission spectrum peaking at 550 nm is observed under low excitation energy of 1 mW/cm^2^. When it increases to 5 mW/cm^2^, a broad and strong emission with the contributions of two components appears. With the increasing excitation power energy, the relative intensity of the low-energy band (614 nm) and high-energy band (550 nm) increases gradually. The emission images of BPOH-TPA powder in excitation energy of 1, 5, and 15 mW/cm^2^ are shown in Figure 3B, showing a clear color change from yellow to orange-red with the CIE coordinates shifting from (0.40, 0.50) to (0.42, 0.43) (Figure 3C). Similar phenomena are also observed in compounds BPOH-PhCz and BPOH-SF. The relative ratios of the two emission bands in the three molecules are calculated and shown in Supplementary Figure S9. It is demonstrated that the higher excitation energy can promote more excitons to the excited state of the E* form, which will transfer to the K* form efficiently via ESIPT, inducing the increase of the low-energy K* emission. Aspired by the dependence of emission colors on the excitation energies, these dual-emissive ESIPT materials show great potential applications in the area of data storage, information encryption, and anti-faking (Demchenko et al., 2013; Zhang et al., 2022).

Furthermore, the dependence of emission spectra on the excitation wavelength at higher excitation energies was investigated. As shown in Figure 3D, dual-emission bands of 525 and 669 nm are clearly observed in BPOH-TPA upon excitation of 300 nm, which can be attributed to the original molecule E* formula and the K* formula arising from ESIPT, respectively. When excited at longer wavelengths, the intensity of the emission band at 525 nm decreases. The high-energy emission band almost disappeared upon the 400 nm excitation. Such distribution of intensities demonstrates the increased ESIPT efficiency with the excitation of lower-energy quanta. In contrast, only low-energy emission bands are observed in molecules BPOH-PhCz and BPOH-SF, which are independent of the excitation wavelength (Supplementary Figures S10, S11). This may ascribe to the electron donating abilities of the donor moiety in these ESIPT molecules.

### Single-Crystal Analyses

**FIGURE 4:**
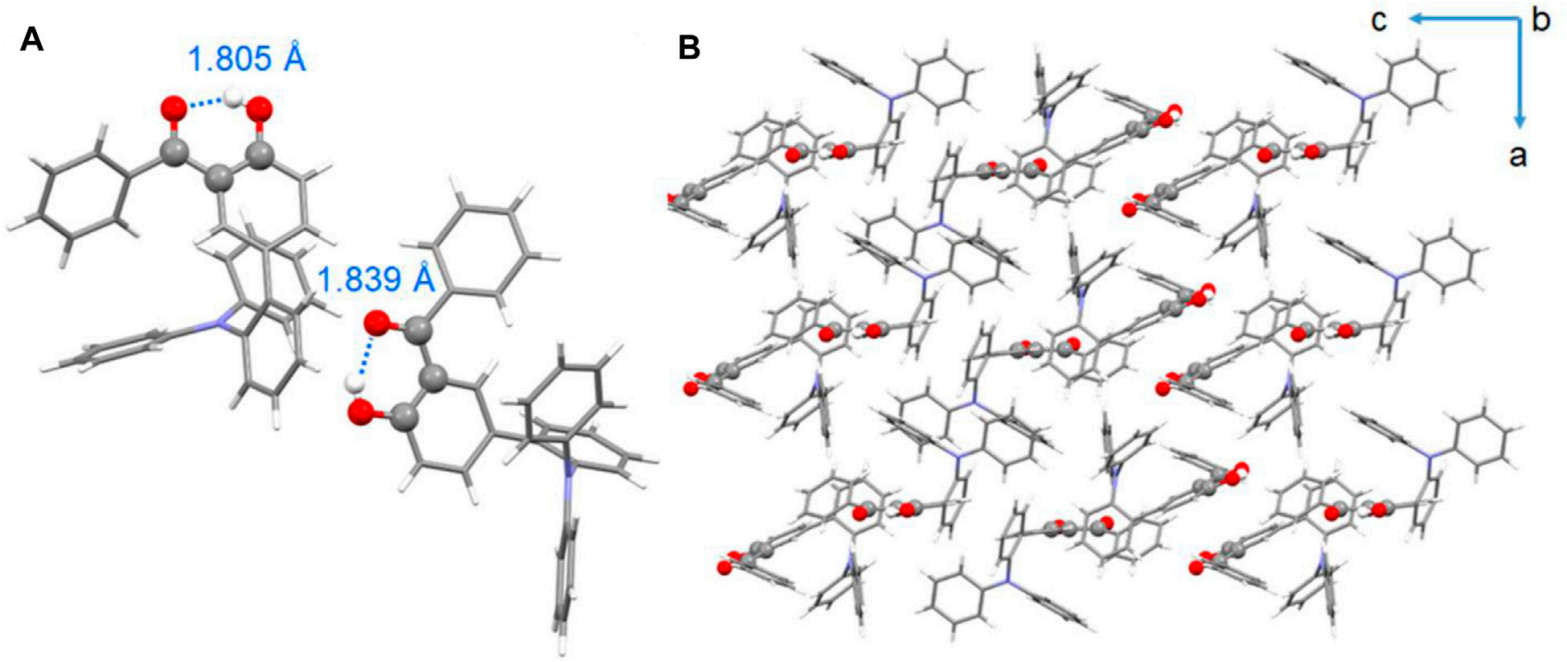
**(A)** Single-crystal structures of BPOH-TPA (C−H···O intramolecular hydrogen bonds are labeled in blue) and **(B)** packing mode of BPOH-TPA from b axis view.

To further verify the ESIPT emission mechanism and investigate the conformational structures, single-crystal X-ray diffraction (XRD) analyses of compounds BPOH-TPA and BPOH-SF were performed. However, due to the possible large steric hindrance of the phenylcarbazole substituent, the growth of crystals suitable for X-ray crystallography using BPOH-PhCz was not possible. Single crystal of BPOH-TPA was achieved by recrystallization from a mixed solvent system by the solvent evaporation method. The CCDC number of the single-crystal structure is 2,078,023 and the data for bond angles and distances are listed in Supplementary Tables S1, S2. It is revealed that the BPOH-TPA crystal is based on the P-1 space group and two types of conformations were detected in the single-crystal structure. For both conformations, BPOH-TPA molecules adopt twisted conformational structures due to the steric hindrance effects. Strong intramolecular O−H···O hydrogen bonds in enol and keto forms with the distances of 1.805 Å and 1.839 Å are clearly observed, respectively (Figure 4A). The O−H···O hydrogen bonds are formed between the oxygen atom of the carbonyl moieties and the O−H groups of the neighboring phenyl ring. Therefore, the protons can be easily transferred through the O−H···O hydrogen bonds after being excited and keto forms of BPOH-TPA could be generated. Thus, excitons in excited states are possible to decay from the lower-energy ESIPT channels to dual-emissive properties. The single-crystal analysis of BPOH-TPA also indicates that the molecules are packing loosely, as shown in Figure 4B, so the molecules are easy to switch the conformation between the E* and K* forms with proton rearrangement through hydrogen bonds. Based on the single-crystal analyses, the lower-energy emission bands for these compounds were further demonstrated as the ESIPT emissions. Compared with BPOH-TPA, the enol form of BPOH-SF shows a longer intramolecular O−H···O distance of 1.852 Å (Supplementary Figure S4), further confirming the relatively weak ESIPT process in BPOH-SF.

## Conclusion

To summarize, a series of ESIPT materials, BPOH-TPA, BPOH-PhCz, and BPOH-SF, were successfully designed, synthesized, and characterized in this work. Molecule BPOH-TPA exhibits dual-emission properties in solution and solid state. Single-crystal analyses and photophysical studies were carried out to explore the dual-emission properties. The emission bands derived from enol and keto forms are dependent on the excitation wavelength, while the other two molecules BPOH-PhCz and BPOH-SF have no such property. Moreover, a gradual redshift from the high-energy emission band ranging from 550 to 582 nm to a low-energy emission band ranging from 625 to 638 nm is observed in all three molecules when the excitation power energy increases from 1 to 15 mW/cm^2^. This is attributed to the promotion of more excitons from E* to K* forms *via* ESIPT at high excitation energy. The emission of these compounds shows a gradual color change from yellow to orange-red. This study successfully presents a strategy for designing dual-emissive and color-tunable ESIPT molecules. Molecules with excitation energy–dependent emissive characteristics have potential advantages in anti-faking and information encryption application areas.

## Data Availability

The original contributions presented in the study are included in the article/Supplementary Material; further inquiries can be directed to the corresponding author.
