# Supplementary material for: 2-Hydroxybenzophenone Derivatives: ESIPT Fluorophores Based on Switchable Intramolecular Hydrogen Bonds and Excitation Energy–Dependent Emission
Source: Front Chem. 2021 Oct 19;9:766179. doi: 10.3389/fchem.2021.766179 (PMC8560898; doi:10.3389/fchem.2021.766179)
Supplement: Supplementary file 1 [file DataSheet1.docx]

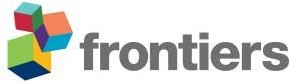


***Supplementary Material***

# Characterization of Compounds


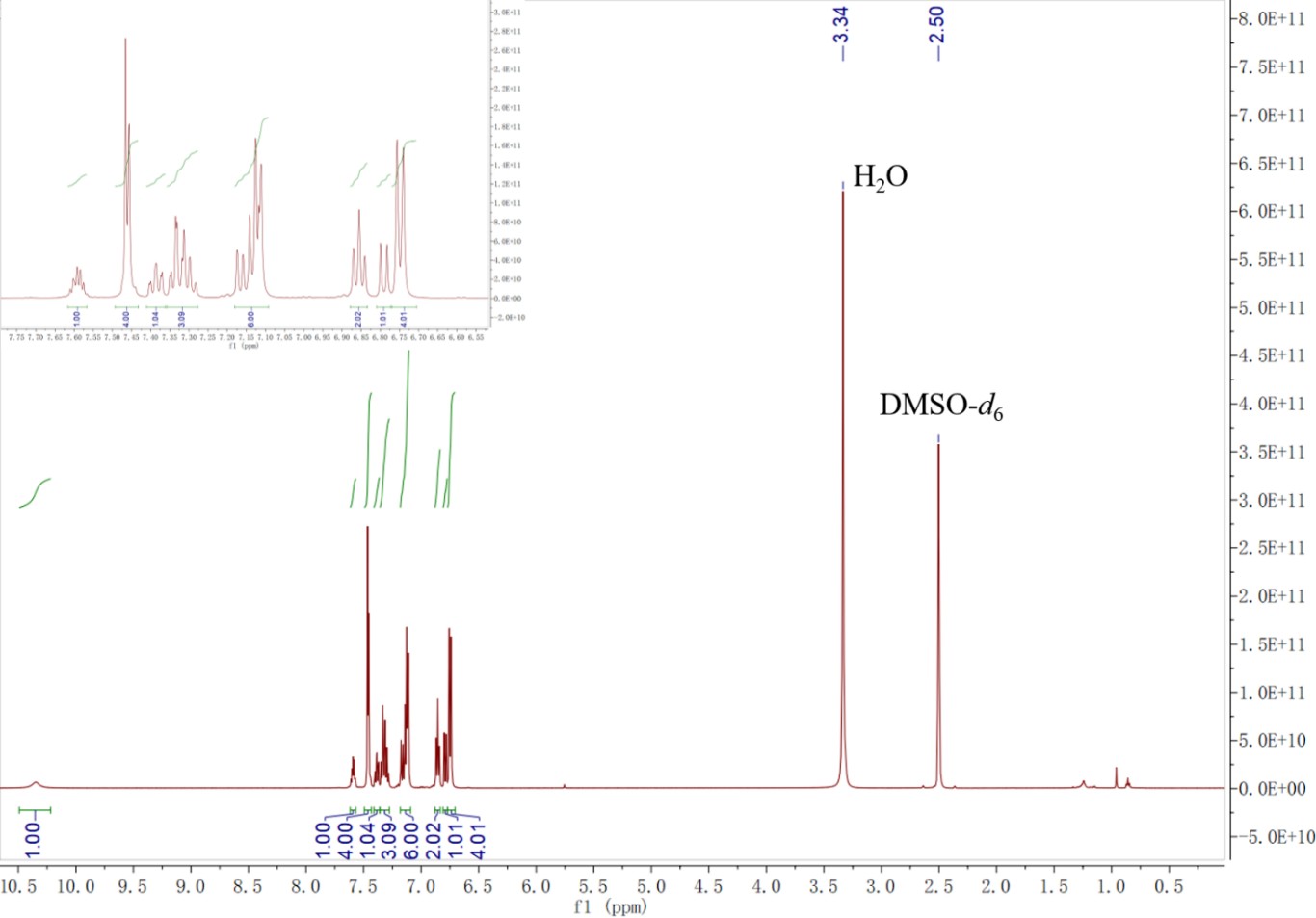


(a)

Supplementary Material


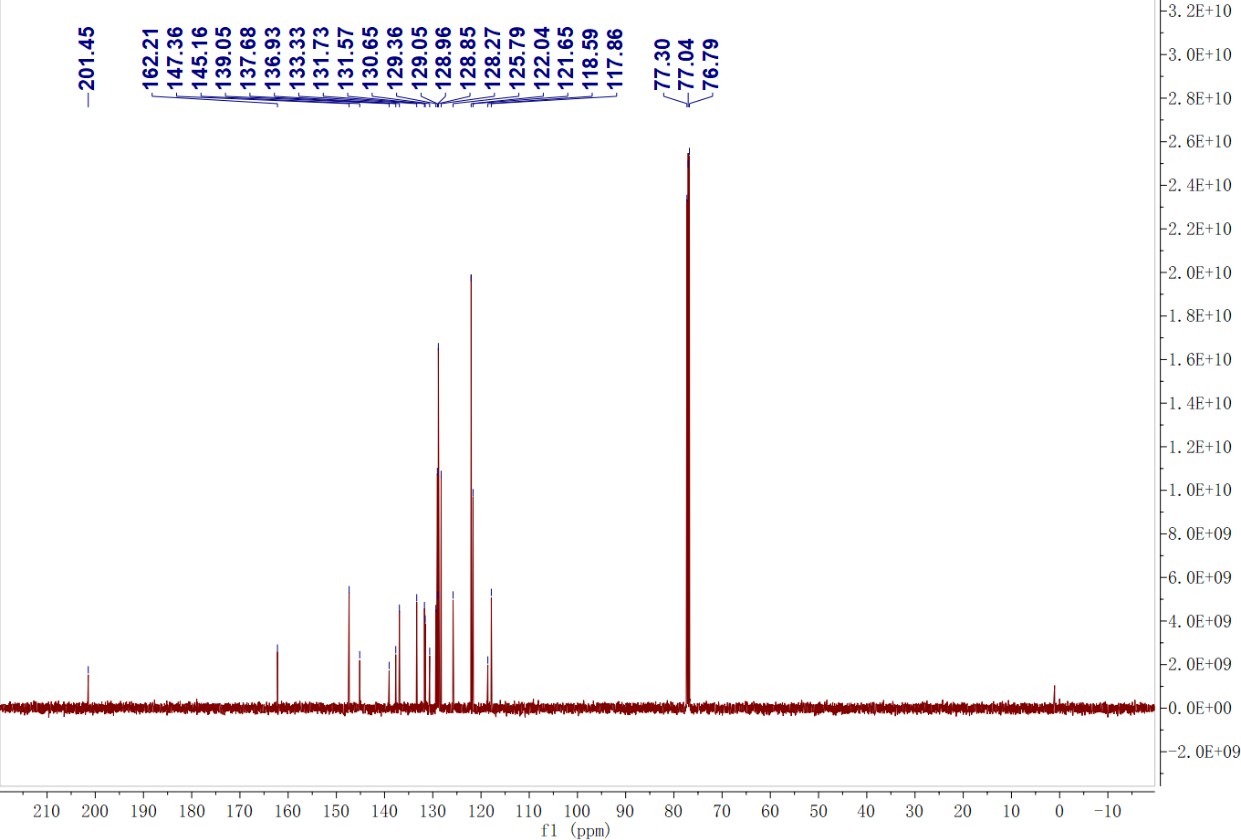


(b)


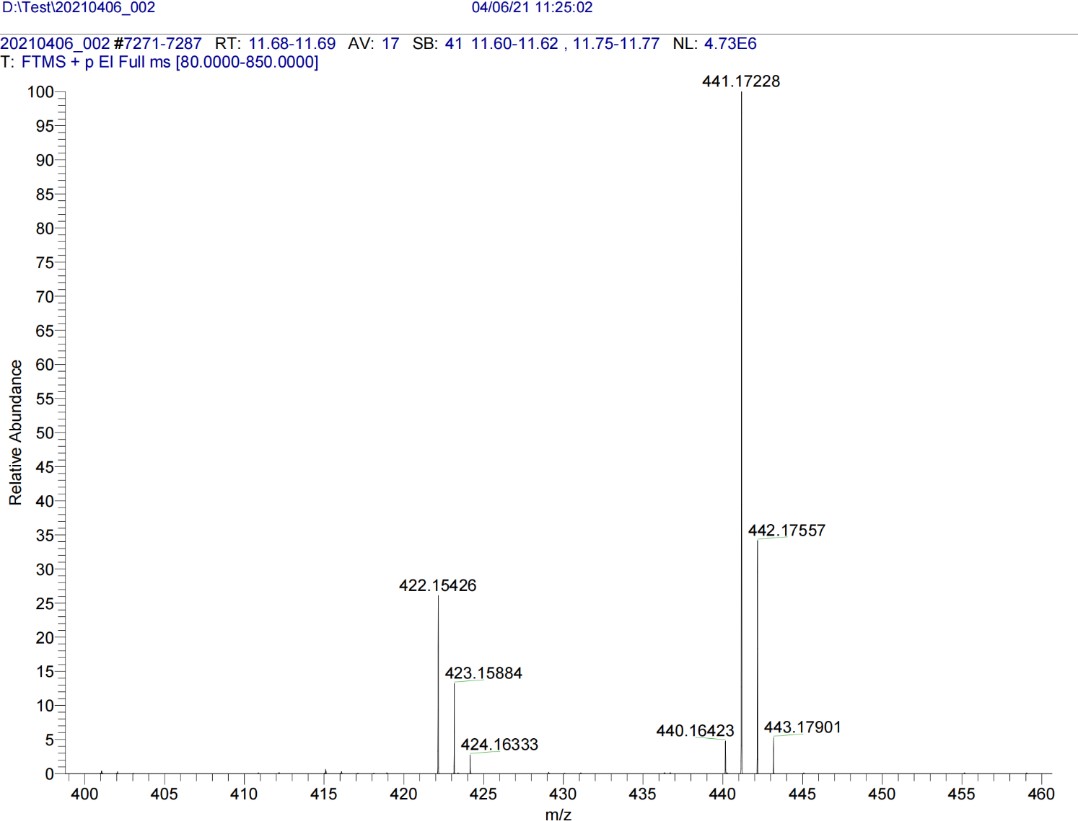


(c)

**Supplementary Figure 1.** (a) 1H-NMR, (b) 13C-NMR and (c) High Resolution EI mass spectra of BPOH-TPA.


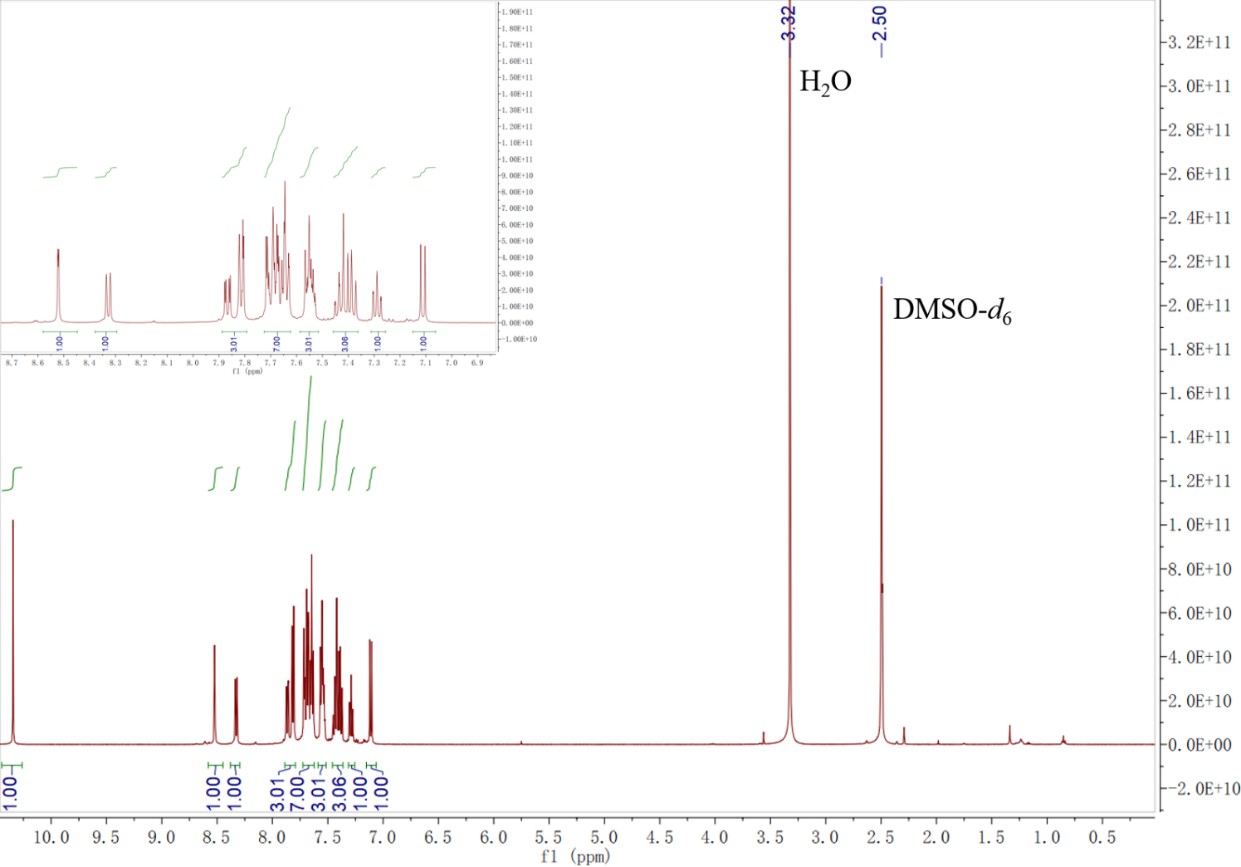


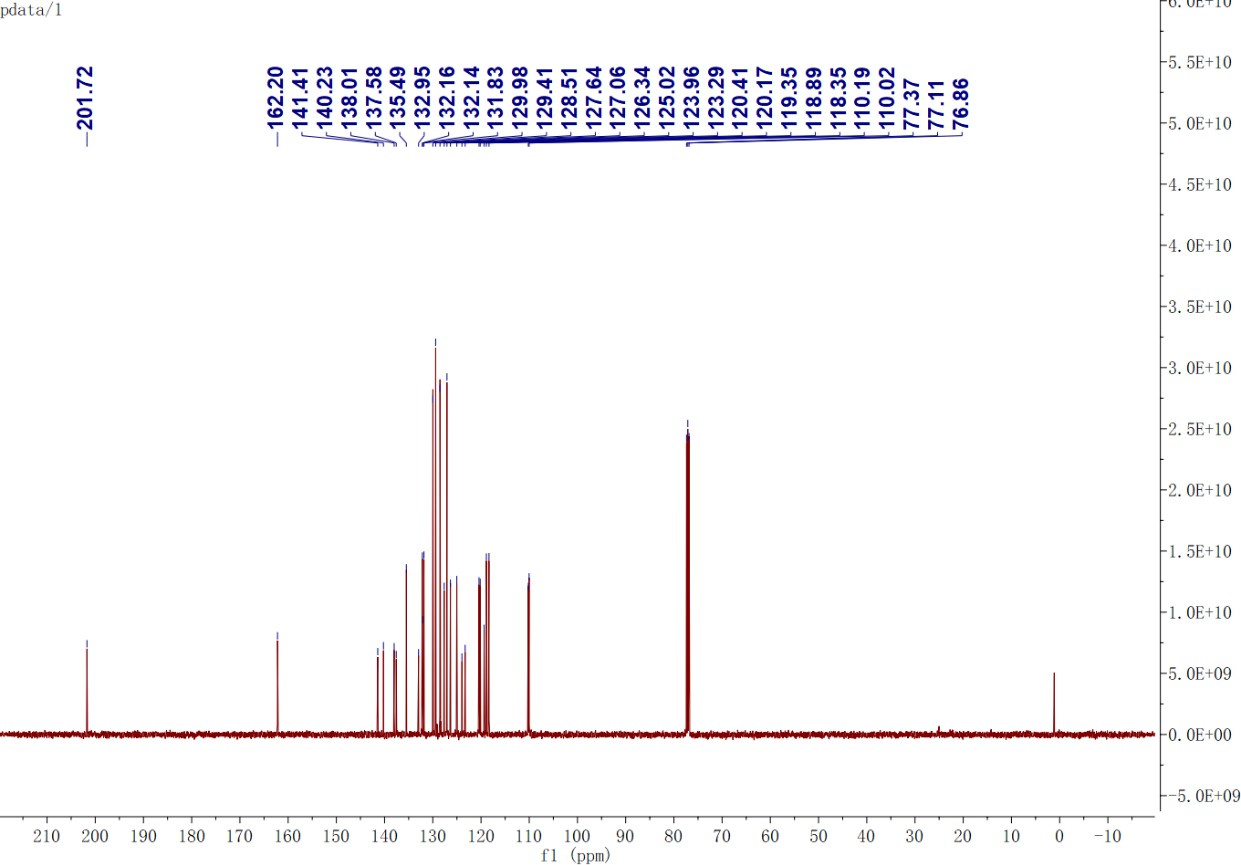
(a)

(b)

Supplementary Material


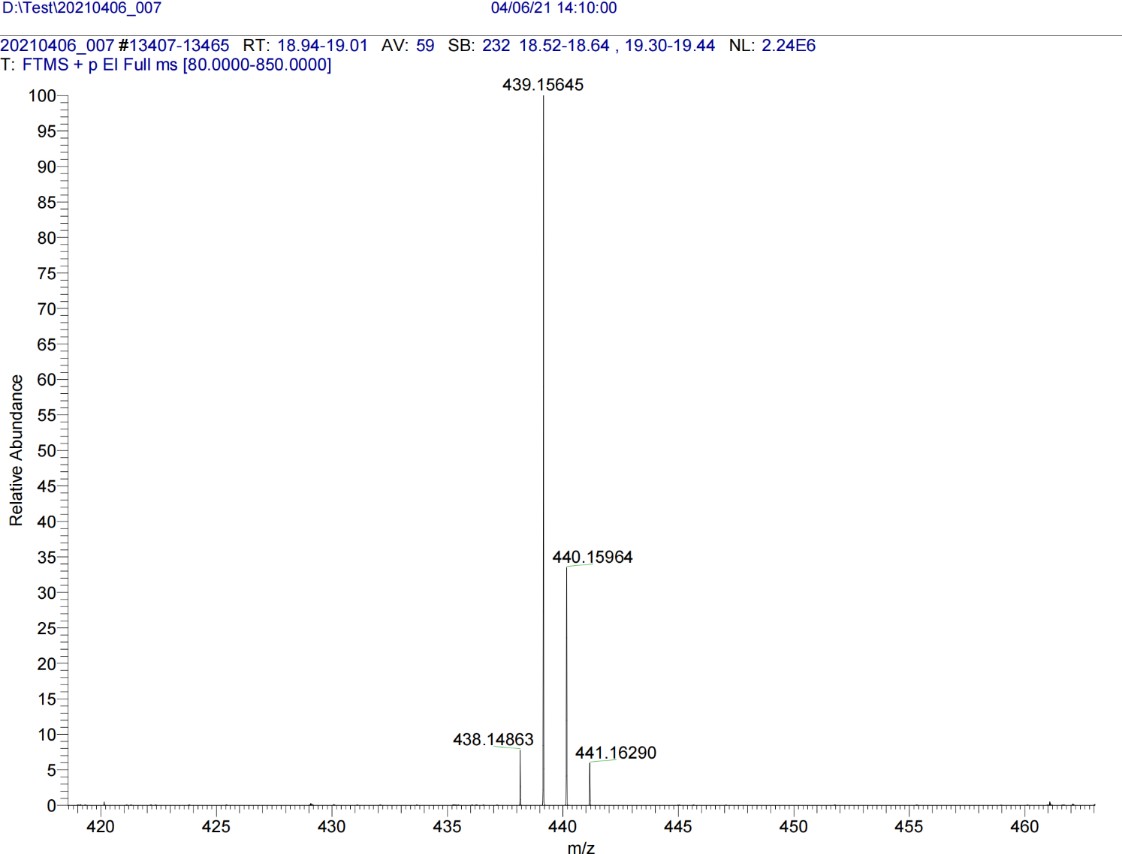


(c)

**Supplementary Figure 2.** (a) 1H-NMR, (b) 13C-NMR and (c) High Resolution EI mass spectra of BPOH-PhCz.


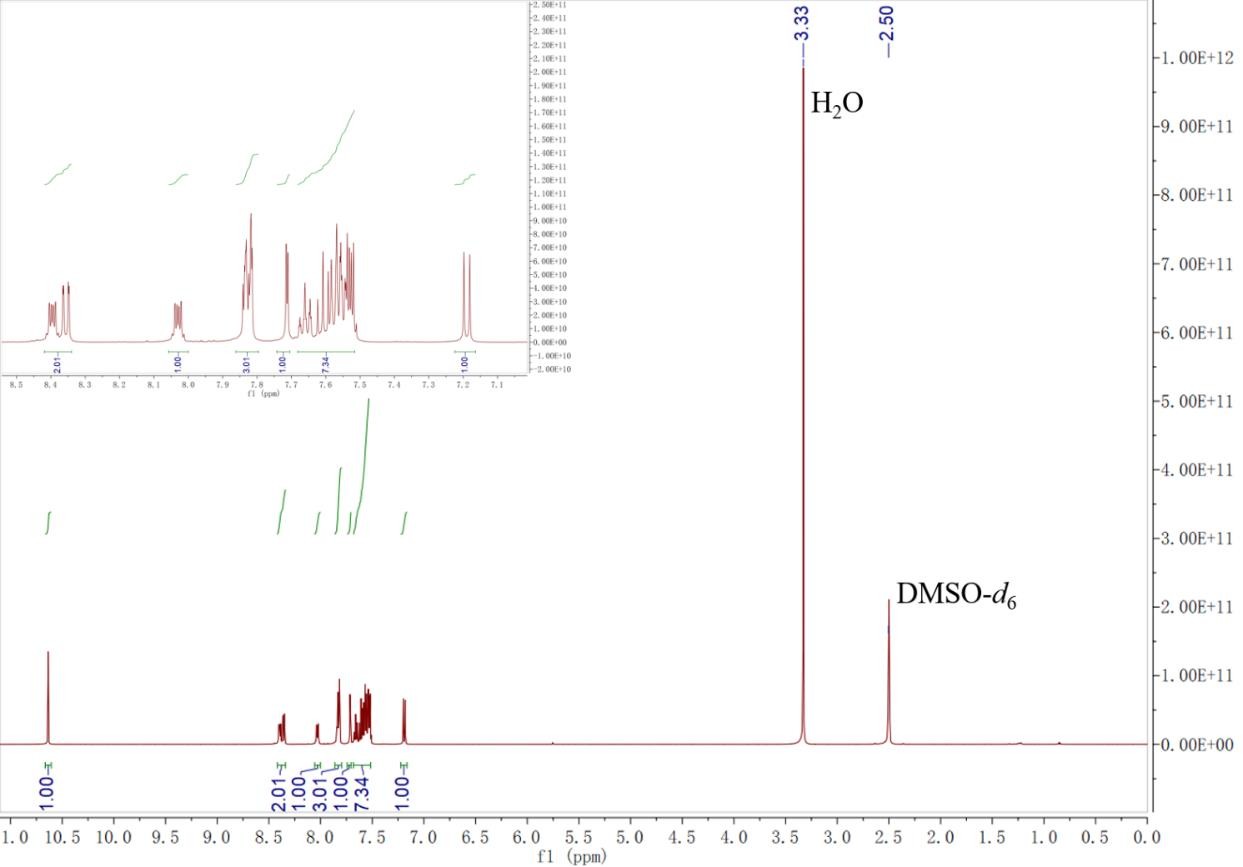


(a)


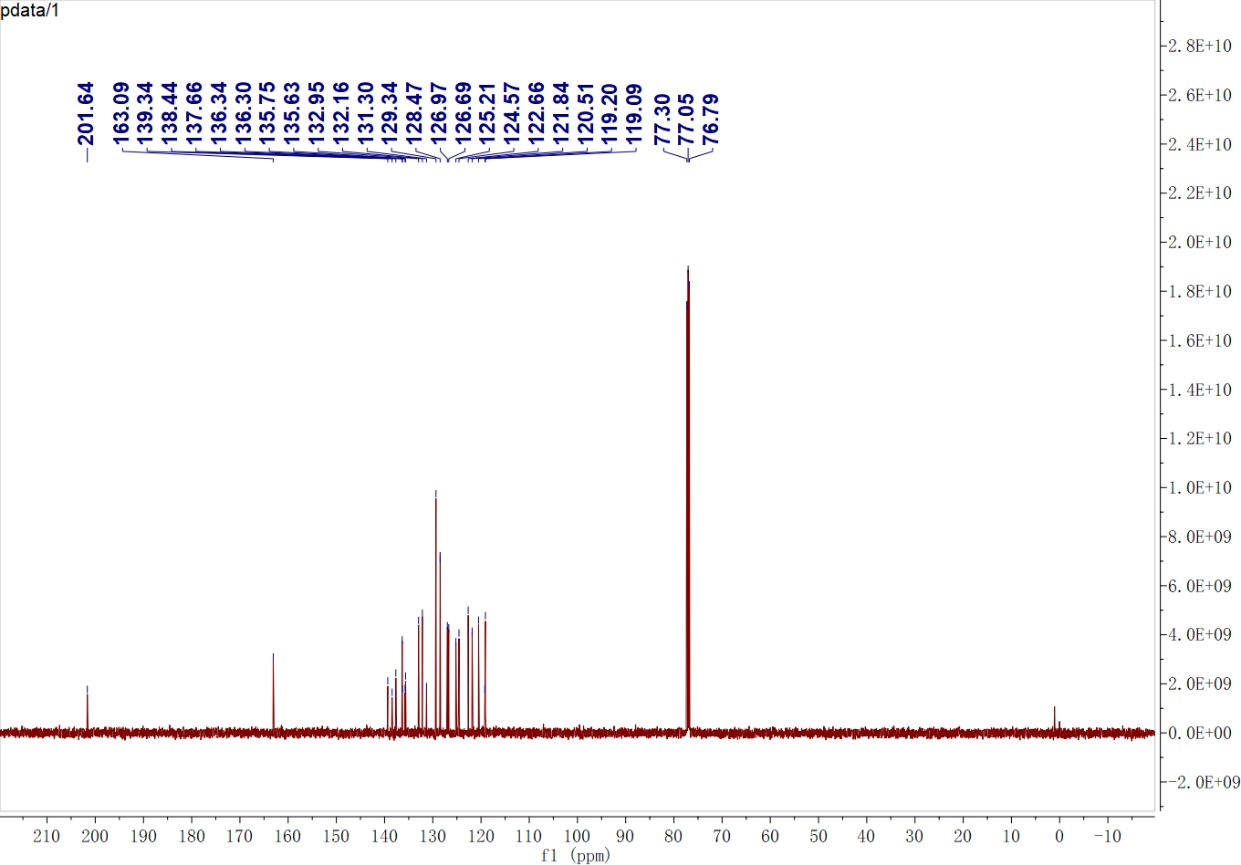


(b)

Supplementary Material


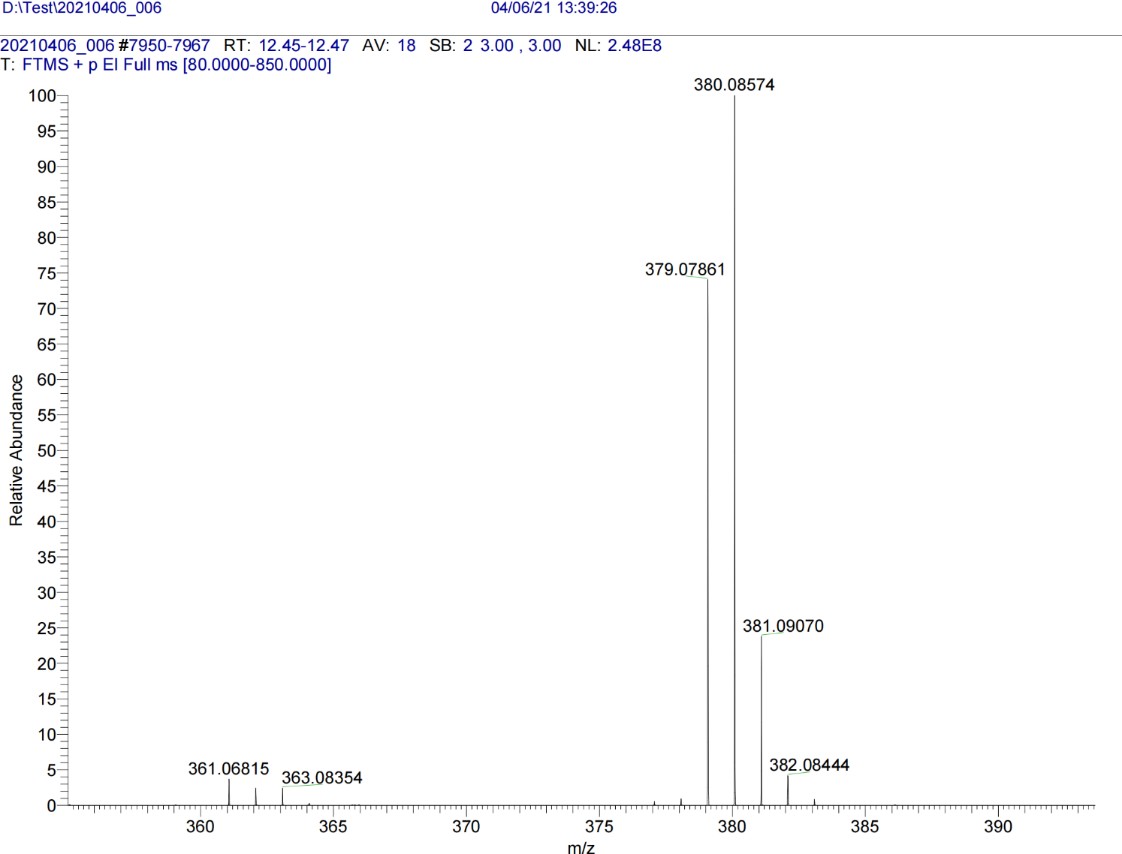


(c)

**Supplementary Figure 3.** (a) 1H-NMR, (b) 13C-NMR and (c) High Resolution EI mass spectra of BPOH-SF.

# Single crystal data of BPOH-TPA

Single-crystal X-ray diffraction data of BPOH-TPA was collected by an Oxford Diffraction Gemini S Ultra X-ray Single Crystal Diffractometer with a (Cu) X-ray source. The single-crystal structures were solved by Olex2 v1.2 program and expanded using Fourier techniques. All non-hydrogen atoms of the compounds were refined with anisotropic thermal parameters. The hydrogen atoms were added in idealized positions and refined with fixed geometry according to their carrier atoms. CCDC numbers for the single-crystal structures of BPOH-TPA are 2078023.

Crystal data for BPOH-TPA: C31H23NO2 (M =441.51 g/mol), triclinic, space group P-1, a = 10.1916(5) Å, b = 10.8178(5) Å, c = 23.6888(11) Å, α=88.0070(10)°, β = 79.6200(10)°, γ=63.4490(10)°, V =

2294.92(19) Å^3^, Z = 2, T = 298(2) K, μ(CuKα) = 0.625 mm^-1^, ρc = 1.278 g/cm^3^, Reflections collected 125679, Independent reflections 8300 unique (Rint = 0.0414, Rsigma = 0.0161). R1= 0.0355 (I>=2σ(I)) and wR2 = 0.0906 (all data). GOF = 1.034.

**Supplementary Table 1**. Bond distances (Å) for BPOH-TPA

| Atom | Atom | Length/Å | Atom | Atom | Length/Å |
| --- | --- | --- | --- | --- | --- |

| O4 | C32 | 1.2430(14) | C38 | C33 | 1.3965(16) |
| --- | --- | --- | --- | --- | --- |
| O3 | C40 | 1.3517(13) | C38 | C37 | 1.3866(17) |
| O2 | C9 | 1.3496(14) | C44 | C39 | 1.4069(15) |
| O1 | C1 | 1.2385(14) | C52 | C53 | 1.3837(16) |
| N2 | C57 | 1.4049(13) | C33 | C32 | 1.4928(17) |
| N2 | C50 | 1.4328(13) | C33 | C34 | 1.3973(17) |
| N2 | C51 | 1.4281(13) | C61 | C60 | 1.3824(16) |
| N1 | C20 | 1.4044(14) | C56 | C55 | 1.3874(16) |
| N1 | C19 | 1.4320(14) | C60 | C59 | 1.3850(16) |
| N1 | C26 | 1.4273(14) | C9 | C10 | 1.3953(17) |
| C57 | C58 | 1.3996(15) | C26 | C31 | 1.3910(17) |
| C57 | C62 | 1.3984(15) | C26 | C27 | 1.3918(17) |
| C50 | C45 | 1.4003(15) | C11 | C10 | 1.3759(17) |
| C50 | C49 | 1.3917(15) | C3 | C4 | 1.3843(17) |
| C58 | C59 | 1.3866(16) | C42 | C41 | 1.3798(17) |
| C45 | C43 | 1.4911(14) | C46 | C47 | 1.3862(16) |
| C45 | C46 | 1.3940(15) | C47 | C48 | 1.3843(17) |
| C62 | C61 | 1.3857(15) | C16 | C17 | 1.3842(17) |
| C13 | C12 | 1.3836(15) | C7 | C6 | 1.3855(17) |
| C13 | C8 | 1.4093(15) | C40 | C39 | 1.4133(16) |
| C2 | C1 | 1.4891(16) | C40 | C41 | 1.3888(18) |

Supplementary Material

| C2 | C3 | 1.3983(15) | C39 | C32 | 1.4742(16) |
| --- | --- | --- | --- | --- | --- |
| C2 | C7 | 1.3968(16) | C37 | C36 | 1.3857(18) |
| C12 | C14 | 1.4887(15) | C53 | C54 | 1.3876(18) |
| C12 | C11 | 1.4053(15) | C18 | C17 | 1.3838(17) |
| C49 | C48 | 1.3845(16) | C25 | C24 | 1.3869(17) |
| C8 | C1 | 1.4765(15) | C21 | C22 | 1.3835(18) |
| C8 | C9 | 1.4126(15) | C4 | C5 | 1.3856(19) |
| C14 | C15 | 1.3959(15) | C31 | C30 | 1.3886(17) |
| C14 | C19 | 1.4008(15) | C55 | C54 | 1.3822(19) |
| C15 | C16 | 1.3866(16) | C27 | C28 | 1.3881(17) |
| C43 | C44 | 1.3829(15) | C24 | C23 | 1.377(2) |
| C43 | C42 | 1.3985(15) | C6 | C5 | 1.3867(18) |
| C51 | C52 | 1.3918(15) | C30 | C29 | 1.3829(19) |
| C51 | C56 | 1.3864(15) | C34 | C35 | 1.381(2) |
| C20 | C25 | 1.3977(16) | C23 | C22 | 1.381(2) |
| C20 | C21 | 1.3942(16) | C36 | C35 | 1.385(2) |
| C19 | C18 | 1.3882(16) | C29 | C28 | 1.3810(19) |

**Supplementary Table 2**. Bond Angles for BPOH-TPA

| Atom | Atom | Atom | Angle/˚ | Atom | Atom | Atom | Angle/˚ |
| --- | --- | --- | --- | --- | --- | --- | --- |

| C57 | N2 | C50 | 120.74(8) | C34 | C33 | C32 | 118.42(10) |
| --- | --- | --- | --- | --- | --- | --- | --- |
| C57 | N2 | C51 | 122.05(8) | C60 | C61 | C62 | 121.30(10) |
| C51 | N2 | C50 | 117.01(8) | C51 | C56 | C55 | 119.93(11) |
| C20 | N1 | C19 | 119.94(9) | C61 | C60 | C59 | 118.82(10) |
| C20 | N1 | C26 | 122.89(9) | O2 | C9 | C8 | 122.73(10) |
| C26 | N1 | C19 | 117.08(9) | O2 | C9 | C10 | 117.40(10) |
| C58 | C57 | N2 | 121.47(9) | C10 | C9 | C8 | 119.85(10) |
| C62 | C57 | N2 | 120.09(9) | C31 | C26 | N1 | 119.53(10) |
| C62 | C57 | C58 | 118.43(9) | C31 | C26 | C27 | 119.41(11) |
| C45 | C50 | N2 | 119.72(9) | C27 | C26 | N1 | 121.05(10) |
| C49 | C50 | N2 | 120.14(9) | C10 | C11 | C12 | 121.14(10) |
| C49 | C50 | C45 | 120.10(9) | C4 | C3 | C2 | 120.03(11) |
| C59 | C58 | C57 | 120.53(10) | C41 | C42 | C43 | 120.78(11) |
| C50 | C45 | C43 | 121.10(9) | C47 | C46 | C45 | 121.09(10) |
| C46 | C45 | C50 | 118.52(10) | C60 | C59 | C58 | 120.76(10) |
| C46 | C45 | C43 | 120.37(10) | C48 | C47 | C46 | 119.95(10) |
| C61 | C62 | C57 | 120.14(10) | C17 | C16 | C15 | 119.82(10) |
| C12 | C13 | C8 | 122.00(10) | C47 | C48 | C49 | 119.78(10) |
| C3 | C2 | C1 | 118.24(10) | C6 | C7 | C2 | 120.01(11) |
| C7 | C2 | C1 | 122.24(10) | O3 | C40 | C39 | 121.90(11) |
| C7 | C2 | C3 | 119.42(10) | O3 | C40 | C41 | 117.72(11) |

Supplementary Material

| C13 | C12 | C14 | 120.66(9) | C41 | C40 | C39 | 120.38(10) |
| --- | --- | --- | --- | --- | --- | --- | --- |
| C13 | C12 | C11 | 118.24(10) | C44 | C39 | C40 | 117.84(10) |
| C11 | C12 | C14 | 121.09(10) | C44 | C39 | C32 | 122.62(10) |
| C48 | C49 | C50 | 120.52(10) | C40 | C39 | C32 | 119.50(10) |
| C13 | C8 | C1 | 122.53(10) | O4 | C32 | C33 | 117.66(10) |
| C13 | C8 | C9 | 118.05(10) | O4 | C32 | C39 | 120.29(11) |
| C9 | C8 | C1 | 119.40(10) | C39 | C32 | C33 | 122.05(9) |
| C15 | C14 | C12 | 120.90(10) | C11 | C10 | C9 | 120.41(10) |
| C15 | C14 | C19 | 118.47(10) | C36 | C37 | C38 | 120.03(11) |
| C19 | C14 | C12 | 120.63(9) | C42 | C41 | C40 | 120.30(11) |
| C16 | C15 | C14 | 121.03(10) | C52 | C53 | C54 | 120.34(11) |
| C44 | C43 | C45 | 120.90(9) | C17 | C18 | C19 | 120.39(11) |
| C44 | C43 | C42 | 118.91(10) | C18 | C17 | C16 | 120.02(11) |
| C42 | C43 | C45 | 120.19(10) | C24 | C25 | C20 | 120.69(11) |
| C52 | C51 | N2 | 121.12(9) | C22 | C21 | C20 | 120.19(12) |
| C56 | C51 | N2 | 119.23(9) | C3 | C4 | C5 | 120.24(11) |
| C56 | C51 | C52 | 119.62(10) | C30 | C31 | C26 | 119.96(11) |
| C25 | C20 | N1 | 120.15(10) | C54 | C55 | C56 | 120.57(11) |
| C21 | C20 | N1 | 121.64(10) | C28 | C27 | C26 | 119.93(12) |
| C21 | C20 | C25 | 118.20(11) | C23 | C24 | C25 | 120.73(12) |

| C14 | C19 | N1 | 119.56(10) | C55 | C54 | C53 | 119.46(11) |
| --- | --- | --- | --- | --- | --- | --- | --- |
| C18 | C19 | N1 | 120.15(10) | C7 | C6 | C5 | 120.22(11) |
| C18 | C19 | C14 | 120.26(10) | C4 | C5 | C6 | 120.03(11) |
| C37 | C38 | C33 | 120.50(11) | C29 | C30 | C31 | 120.61(12) |
| C43 | C44 | C39 | 121.77(10) | C35 | C34 | C33 | 120.30(12) |
| O1 | C1 | C2 | 118.31(10) | C24 | C23 | C22 | 118.79(12) |
| O1 | C1 | C8 | 120.38(10) | C35 | C36 | C37 | 119.82(13) |
| C8 | C1 | C2 | 121.30(9) | C28 | C29 | C30 | 119.39(11) |
| C53 | C52 | C51 | 120.07(11) | C23 | C22 | C21 | 121.38(12) |
| C38 | C33 | C32 | 122.54(10) | C29 | C28 | C27 | 120.69(12) |
| C38 | C33 | C34 | 118.83(11) | C34 | C35 | C36 | 120.49(12) |

# Single crystal data of BPOH-SF

Single-crystal X-ray diffraction data of BPOH-SF was collected by a Bruker Smart APEX II X-ray Single Crystal Diffractometer with a (Cu) X-ray source. The single-crystal structures were solved by Olex2 v1.2 program and expanded using Fourier techniques. All non-hydrogen atoms of the compounds were refined with anisotropic thermal parameters. The hydrogen atoms were added in idealized positions and refined with fixed geometry according to their carrier atoms. CCDC numbers for the single-crystal structures of BPOH-SF are 2108804.

Crystal data for BPOH-SF: C25H16O2S (M =380.4 g/mol), monoclinic, space group P 21/c, a = 17.5853(14) Å, b = 14.1664(12) Å, c = 7.4587(6) Å, α=90°, β = 93.998(1)°, γ=90°, V = 1853.6(3)Å3,

Z = 30, T = 100.15 K, μ(CuKα) = 0.193 mm^-1^, ρc = 1.363 g/cm^3^, Reflections collected 9278, Independent reflections 3255 unique (Rint = 0.0225, Rsigma = 0.0237). R1= 0.0367 and wR2 = 0.0774 (all data). GOF = 1.046.

**Supplementary Table 3**. Bond distances (Å) for BPOH-SF

| Atom | Atom | Length/Å | Atom | Atom | Length/Å |
| --- | --- | --- | --- | --- | --- |
| S1 | C19 | 1.7596(15) | C25 | C24 | 1.401(2) |

Supplementary Material

| S1 | C20 | 1.7505(15) | C13 | C8 | 1.402(2) |
| --- | --- | --- | --- | --- | --- |
| O2 | C9 | 1.3523(18) | C6 | C7 | 1.495(2) |
| O1 | C7 | 1.2415(18) | C6 | C5 | 1.397(2) |
| C14 | C12 | 1.485(2) | C6 | C1 | 1.394(2) |
| C14 | C19 | 1.406(2) | C7 | C8 | 1.473(2) |
| C14 | C15 | 1.399(2) | C11 | C10 | 1.382(2) |
| C9 | C8 | 1.413(2) | C15 | C16 | 1.393(2) |
| C9 | C10 | 1.392(2) | C20 | C21 | 1.397(2) |
| C12 | C13 | 1.385(2) | C5 | C4 | 1.386(2) |
| C12 | C11 | 1.406(2) | C21 | C22 | 1.382(2) |
| C17 | C18 | 1.396(2) | C24 | C23 | 1.382(2) |
| C17 | C16 | 1.379(2) | C1 | C2 | 1.389(2) |
| C18 | C25 | 1.452(2) | C4 | C3 | 1.391(2) |
| C18 | C19 | 1.408(2) | C23 | C22 | 1.399(2) |
| C25 | C20 | 1.406(2) | C3 | C2 | 1.386(2) |

**Supplementary Table 4**. Bond Angles for BPOH-SF

| Atom | Atom | Atom | Angle/˚ | Atom | Atom | Atom | Angle/˚ |
| --- | --- | --- | --- | --- | --- | --- | --- |
| C20 | S1 | C19 | 91.32(7) | C1 | C6 | C5 | 119.88(14) |
| C19 | C14 | C12 | 123.57(13) | O1 | C7 | C6 | 118.64(13) |
| C15 | C14 | C12 | 119.89(13) | O1 | C7 | C8 | 120.89(14) |

| C15 | C14 | C19 | 116.53(13) | C8 | C7 | C6 | 120.46(13) |
| --- | --- | --- | --- | --- | --- | --- | --- |
| O2 | C9 | C8 | 122.37(13) | C9 | C8 | C7 | 119.99(13) |
| O2 | C9 | C10 | 118.09(13) | C13 | C8 | C9 | 118.36(13) |
| C10 | C9 | C8 | 119.55(13) | C13 | C8 | C7 | 121.58(14) |
| C13 | C12 | C14 | 119.73(13) | C10 | C11 | C12 | 121.21(14) |
| C13 | C12 | C11 | 117.67(13) | C16 | C15 | C14 | 122.10(14) |
| C11 | C12 | C14 | 122.56(13) | C11 | C10 | C9 | 120.59(14) |
| C16 | C17 | C18 | 119.26(14) | C25 | C20 | S1 | 112.41(11) |
| C17 | C18 | C25 | 128.16(13) | C21 | C20 | S1 | 126.00(12) |
| C17 | C18 | C19 | 119.74(13) | C21 | C20 | C25 | 121.59(14) |
| C19 | C18 | C25 | 112.08(13) | C4 | C5 | C6 | 119.91(15) |
| C20 | C25 | C18 | 112.10(13) | C17 | C16 | C15 | 120.64(14) |
| C24 | C25 | C18 | 128.99(14) | C22 | C21 | C20 | 118.19(14) |
| C24 | C25 | C20 | 118.90(14) | C23 | C24 | C25 | 119.63(15) |
| C14 | C19 | S1 | 126.23(11) | C2 | C1 | C6 | 119.79(15) |
| C14 | C19 | C18 | 121.70(13) | C5 | C4 | C3 | 120.08(16) |
| C18 | C19 | S1 | 112.02(11) | C24 | C23 | C22 | 120.60(14) |
| C12 | C13 | C8 | 122.55(14) | C21 | C22 | C23 | 121.04(14) |
| C5 | C6 | C7 | 121.50(14) | C2 | C3 | C4 | 120.06(15) |
| C1 | C6 | C7 | 118.55(14) | C3 | C2 | C1 | 120.25(15) |

Supplementary Material


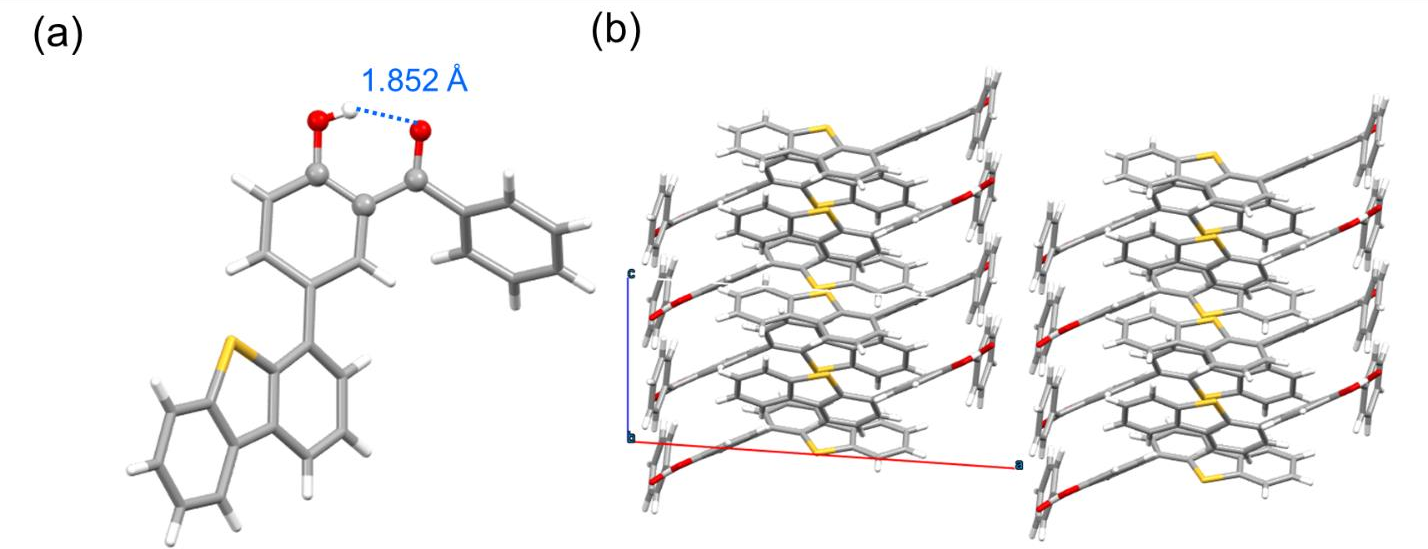


**Supplementary Figure 4.** (a) Single crystal structures of BPOH-SF (C−H···O intramolecular hydrogen bonds are labeled in blue), (b) packing mode of BPOH-SF from b axis view.

# Theoretical calculation

Molecular geometries were extracted and performed by Gaussian 09W program package with time- dependent density functional theory (TD-DFT) with Beck's three-parameter hybrid exchange functional and Lee (Mao et al., 2019), and Yang and Parr correlation functional (B3LYP) (Yang et al., 2020) with 6-311G* basic set.

In molecule BPOH-TPA, the calculated energies of the lowest singlet excited states in enol and keto forms are 2.651 and 2.632 eV, respectively. The extremely small energy gap of 0.02 eV between the two tautomers confirms the fast ESIPT process. When the excitation power energy is further increase, this process can be further enhanced. Therefore, more excitons can be transferred and accumulated in the keto form, inducing the clear color change.

# Photophysical data and spectra


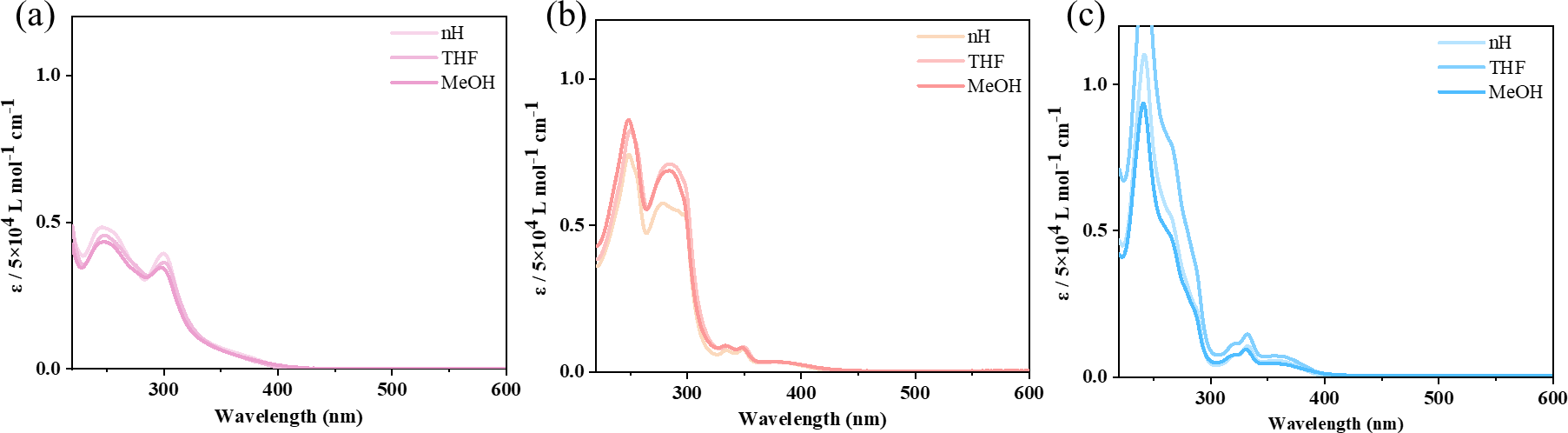


**Supplementary Figure 5.** Absorption spectra of a) BPOH-TPA, b) BPOH-PhCz and c) BPOH-SF in different solutions **(**nH = n-hexane, THF = Tetrahydrofuran, MeOH = Methanol, 2.0 × 10−5 mol L−1**)**.

10^2^


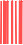

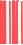


BPOH-TPA emission at 405 nm

τ=3.26 ns

Intensity (Counts)

10^1^

0 10 20 30 40 50

Time (ns)

**Supplementary Figure 6.** Lifetime decay profile of BPOH-TPA at 405 nm in THF (2 × 10−5 mol L−1) at room temperature.

10^2^


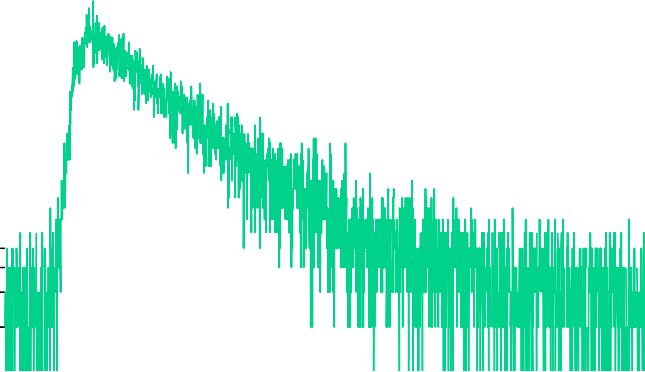


BPOH-PhCz emission at 343 nm

τ=7.38 ns

Intensity (Counts)

10^1^

0 10 20 30 40 50

Time (ns)

**Supplementary Figure 7.** Lifetime decay profile of BPOH-PhCz at 343 nm in THF (2 × 10−5 mol L−1) at room temperature.

Supplementary Material

10^2^


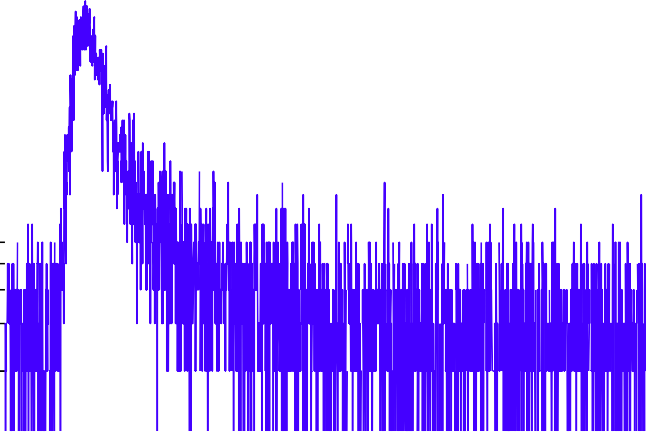


BPOH-SF emission at 382 nm

τ=2.31 ns

10^1^

Intensity (Counts)

0 10 20 30 40 50

Time (ns)

**Supplementary Figure 8.** Lifetime decay profile of BPOH-SF at 382 nm in THF (2 × 10−5 mol L−1) at room temperature.

1.4

BPOH-TPA BPOH-PhCz BPOH-SF

1.2

Low-energy/high-energy

1.0

0.8

0.6

0 2 4 6 8 10 12 14 16

Excitation energy (mW/cm^2^)

**Supplementary Figure 9.** The ratios of the low- and high-energy bands of the ESIPT emission bands with the different excitation energy, where low-energy and high-energy represent the normalized intensity of the low-energy and high-energy bands, respectively.

**Supplementary Figure 10.** The dependence of steady-state emission spectra of BPOH-PhCz powder on the excitation wavelength. Excitation power at around 60 mW/cm2.

**Supplementary Figure 11.** The dependence of steady-state emission spectra of BPOH-SF powder on the excitation wavelength. Excitation power at around 60 mW/cm2.

# Reference

Mao Z, Yang Z, Xu C, Xie Z, Jiang L, Gu F L, et al, (2019). Two-photon-excited ultralong organic room temperature phosphorescence by dual-channel triplet harvesting. *Chem. Sci*. 10, 7352-7357. doi: 10.1039/c9sc02282a.

Supplementary Material

Yang Z, Xu C, Li W, Mao Z, Ge X, Huang Q, et al. (2020). Boosting the Quantum Efficiency of Ultralong Organic Phosphorescence up to 52 % via Intramolecular Halogen Bonding. *Angew. Chem. Int. Ed.* 59, 17451-17455. doi: 10.1002/anie.202007343.
